# Learning curve in relation to radiation exposure, procedure duration and complications rate for Minimally Invasive Chevron Akin (MICA) osteotomy

**DOI:** 10.1186/s12891-023-06706-1

**Published:** 2023-07-15

**Authors:** Matjaž Merc, Samo Karel Fokter, Ibad Sha I

**Affiliations:** Department of Orthopaedics, University Clinical Centre Maribor, Ljubljanska Ulica 5, 2000 Maribor, Slovenia

**Keywords:** Operation time, Fluoroscopy, Hallux valgus, Bunion correction, Percutaneous, Minimally invasive surgery

## Abstract

**Background:**

Minimally invasive chevron Akin osteotomy (MICA) has become increasingly common and is compatible with the traditional open approaches for hallux valgus correction. However, it is impeded by concerns regarding the steep learning curve, increased radiation exposure and some specific complications. No standardized method for identifying a learning curve exists. We used a reproducible mathematical model to accurately define the learning curve of MICA with a focus on fluoroscopy time, procedure duration and complications rate.

**Methods:**

We conducted a retrospective study of MICA procedure performed by a single surgeon during his initial experience with this procedure. The chronologic case number was plotted against variables of interest and learning was identified as the point at which instantaneous rate of change of a curve fit to the data set equalled the average rate of change of the data set.

**Results:**

One hundred cases have been analysed. Learning plateau in operation time was achieved after 29 cases, with the first 29 cases requiring a median of 60 min compared to 40 min for the latter 71 cases. Proficiency in fluoroscopy application occurred in case 30. The median fluoroscopy time for the first 30 cases was 86 seconds compared to 70 seconds in another 70 cases. The complication rate plateau was reached after 42 cases, with 15 of 22 complications occurring in the group operated first.

**Conclusion:**

Results demonstrate surgeon’s comfort with MICA to minimize operative time and radiation exposure after 30 cases. The plateau is achieved later for complications. Findings impose lag between surgeon feeling comfortable with procedure and a decrease in complications. Further research is reasonable to analyse several surgeons learning curve and to generate a potential reference learning curve that could serve as a normative.

**Trial registration:**

UKC-MB-KME-33/19, retrospectively registered.

## Introduction

Hallux Valgus (HV) is one of the commonest foot disorders with a prevalence of up to 35.7% in population aged above 65 years and is associated with impaired balance, increased risk of falling, disability and lower quality of daily life [1–3]. Once symptomatic, operative treatment is considered optimal and over the years more than at least 150 different surgeries has been described in the literature with no enthralling evidence to recommend any specific technique [4].

Recently, minimally invasive surgery (MIS) is increasing in popularity, especially minimally invasive chevron Akin osteotomy (MICA) for its advantages of reduced morbidity, smaller scars, soft tissue preservation, faster recovery and rehabilitation times while achieving outcomes comparable with those of traditional open osteotomy approaches [4–6]. MICA is the third generation MIS with first generation being percutaneous distal metatarsal osteotomy without internal fixation while in the second generation Kirschner wire fixation is done [5, 6]. The first and second generations were associated with a range of serious complications, including pseudarthrosis and malunion [7, 8].

Even though MICA has many added benefits, the procedure has a learning curve which ranges from approximately 20 to 50 cases and also a fluoroscopic guidance is necessary most of the time throughout the procedure [9, 10]. Usually fluoroscopy is not routinely used during open HV correction, hence radiation exposure in MICA presents a risk to the surgeon, the patient, and the entire operating room staff. Multiple studies in the literature have shown that operating time, complication rates, and patient outcomes all improve as the surgeon gains experience [11, 12]. However, an examination of fluoroscopy time in MICA procedure as a parameter of surgeon’s learning curve has not been studied previously. Furthermore, a systemic mathematical model as well as a standardized method to accurately define and identify the learning curve doesn't exist yet, making it difficult to interpret the results of multiple different learning curve studies. In this study, we use a reproducible systemic mathematical model to accurately define the learning curve of MICA procedure with a focus on operation time, fluoroscopy time and complications rate and their association with surgeon's learning curve.

## Materials and methods

This is retrospective study of single surgeons’ performance analysed on consecutive MICA procedures in one institution from 2017 to the end of 2019. The surgeon is a dedicated foot and ankle specialist with no experience in MICA surgery. Before starting MICA, he has been comfortable with non-minimally invasive hallux valgus correction techniques. When started doing MICA and other foot related minimally invasive procedures he, attended basic and advanced Foot & Ankle MIS Cadaver Lab organized by GRECMIP (MIFAS) group in 2017. Therefore, this series represents the surgeon’s initial experience of adopting minimally invasive techniques in hallux valgus correction. The procedure was performed with one assistant that was a resident or a medical student with no prior HV surgery experience. Operation procedure, duration and demographic data were obtained via the electronic medical record. Duration of surgery corresponds to the time span between the skin incision and final wound dressing. Fluoroscopy time and exposure were obtained from Flouroscan InSight 2 imaging device (Hologic, Marlborough, MA, USA) that has been used for all procedures. Postoperative complications were identified via outpatient clinic follow-up notes and subsequent operative reports.

### Indication

The severity of the deformity was categorized according to severity into three grades: mild (Hallus Valgus Angle (HVA) < 20°, Inter-Metatatarsal Angle (IMA) 9–11°), moderate (HVA 20–40°, IMA 12–16°) and severe (HVA > 40°, IMA > 16°) and an indication for MICA procedure was symptomatic mild to severe HV deformity with no evident signs of the first MTP joint arthrosis or first ray hypermobility. The patients with any further deformities requiring simultaneous correction, including those who needed second toe correction, were excluded from this analysis.

### Procedure

**Fig. 1:**
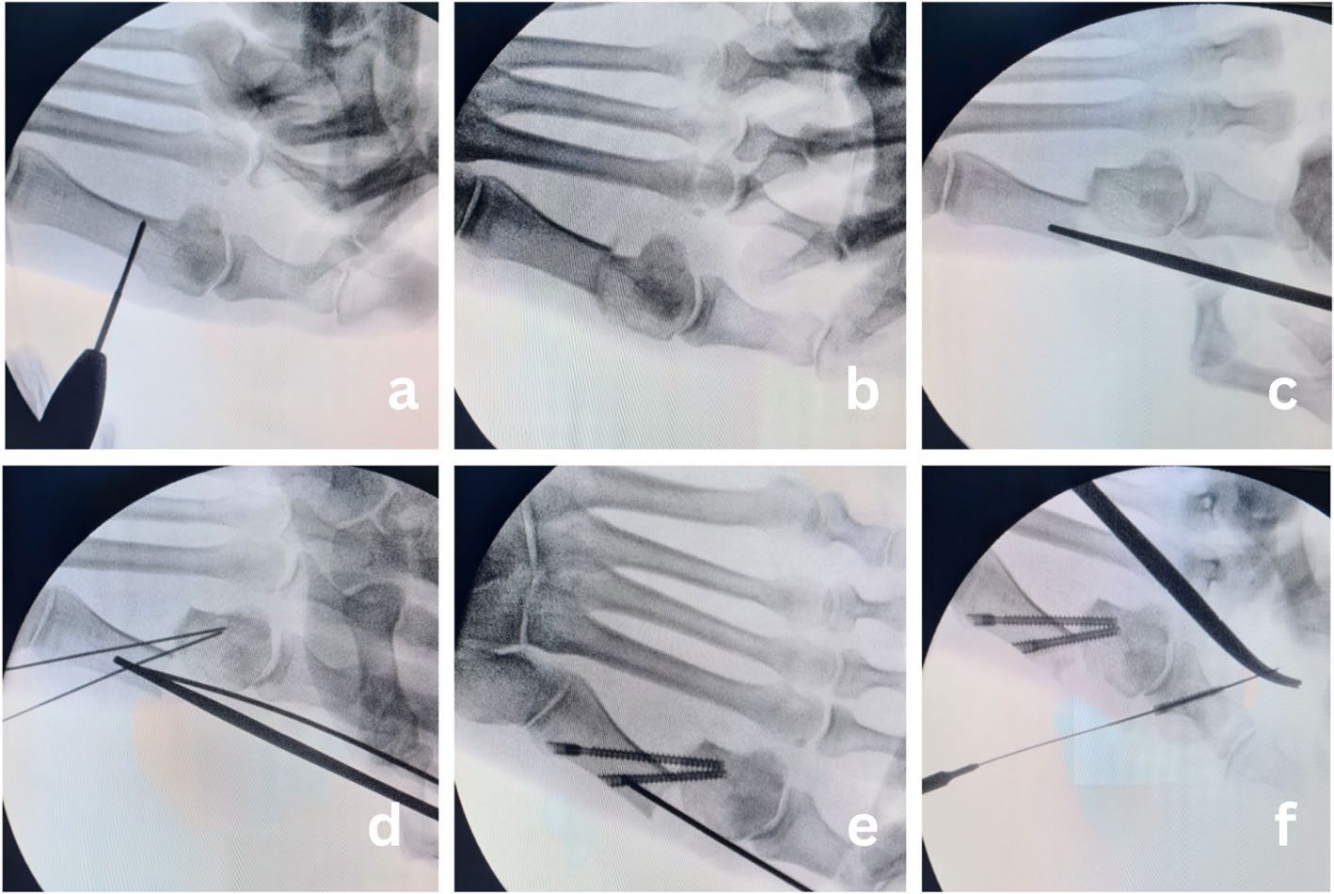
Intraoperative sequence showing surgical technique; **a**) Insertion of the burr **b**) Completed osteotomy **c**) Displacement of the metatarsal head with Kocher’s forceps **d**) Drilling of guidewires in desired position **e**) Fixation of second screw along with the first screw in position. **f**) Akin osteotomy and fixation

**Fig. 2:**
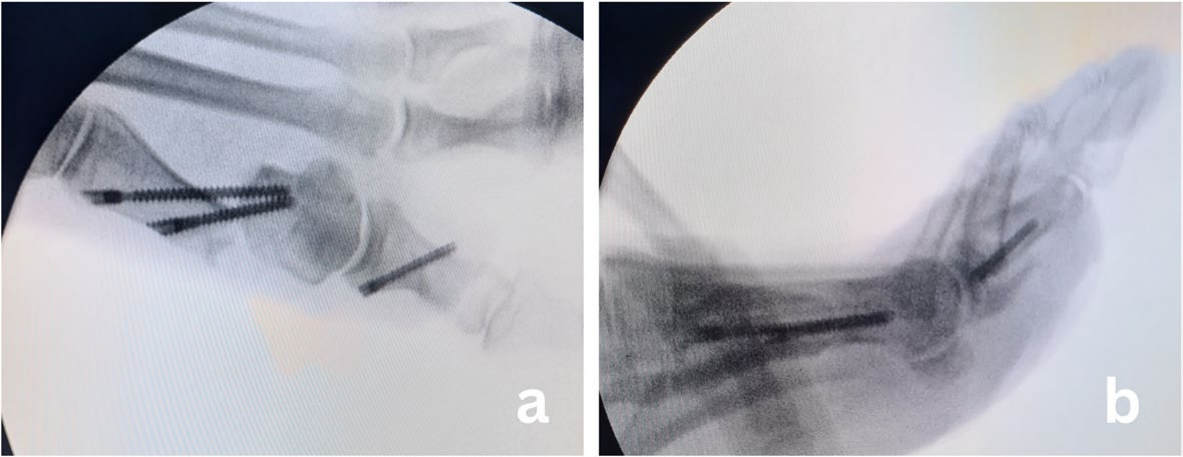
Final fixation. **a** Anteroposterior view **b**) Lateral view

**Fig. 3:**
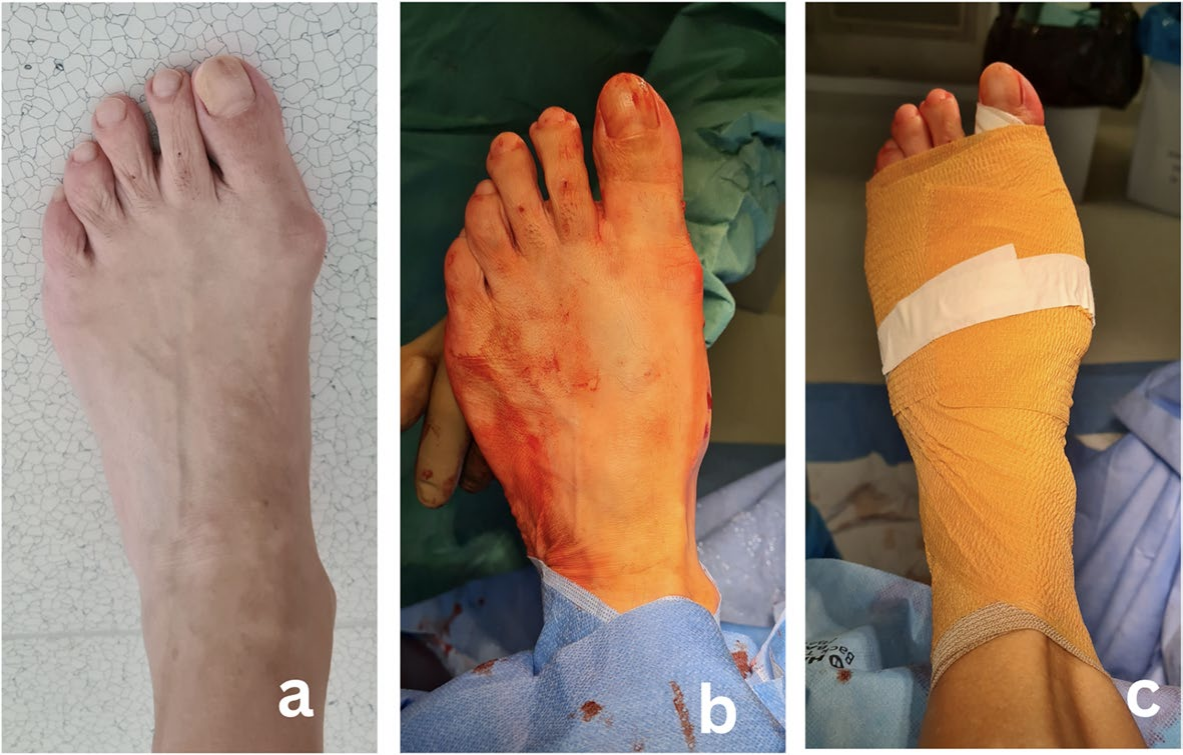
Postoperative result and dressing application. **a** Preoperative hallux valgus **b**) Correction after MICA procedure **c**) Special dressing supports deformity correction and serves as immobilisation

The procedure was performed under general or regional anaesthesia. No tourniquet was used. The foot was placed over the end of the table to allow intraoperative fluoroscopy. With a No. 15 surgical blade, a 3 mm incision was made medially just proximal to the medial eminence. The chevron osteotomy was created with a burr of 2.2 mm diameter and 20 mm in length (Fig. 1a). The burr was introduced at the level of the neck of the metatarsal and a "V" shape cut of the bone was performed, pointed in the desired direction under fluoroscopic guidance. A Kocher forceps was then inserted through the incision into the proximal diaphyseal channel and was used as a lever to translate the metatarsal head (Fig. 1b). Under fluoroscopic guidance, the head was translated laterally to achieve deformity correction and then temporary fixed with K-wire that replaced the Kocher forceps. Afterwards, the metatarsal head was internally fixed with two 4 mm cannulated cancellous percutaneous screws (Mi-screw, CHOC, Montauban, France) (Fig. 1c-e). Once chevron osteotomy was completed, percutaneous Akin (medial closing wedge osteotomy) procedure of first phalanx was done with the same burr as for chevron osteotomy and internally fixed with 2.5 mm HCS screw (Zimmer Biomet, Warsaw, IN, USA) (Fig. 1f). No percutaneous release of the lateral MTP joint capsule was performed. Intraoperative radiographs were taken to confirm osteotomy position, direction, satisfactory fixation of the osteotomy as well as to confirm the adequacy of deformity correction (Fig. 2). At the end of the procedure, a specific dressing was applied that also served as a redressive bandage (Fig. 3).

### Statistical analysis

All statistical analyses were done using SPSS ver. 26.0 (SPSS Inc., IL, USA). The identification of the learning curve began with testing the correlation between each variable and sequential case number. When a statistically significant correlation was found, a linear slope was fit to the overall data set, giving an average rate of change for all cases. This step provides a quantifiable average improvement in a variable of interest, such as operative time, per case. A nonlinear association (learning) curve was then fit to the data set by applying an estimation regression model that offered a high R-value and was consistent with the actual situation. The derivate of the equation for this nonlinear curve was solved to find the case at which point slope of the curve equalled the linear slope. This identified case thus equals the point at which the average rate of change on the linear curve equals the instantaneous rate of change on the nonlinear curve. Therefore, the rate of change after this case will always be less than the average rate of change, suggesting that a plateau in learning had occurred. To solve the learning curve for a dichotomous outcome, such as complication rates, cumulative complication number was plotted against time. All complications, whether related to the malunion, loss of fixation, hardware removal, stiffness, or infection, were included in the cumulative complication number because all these factors are positively impacted by surgeon experience, that is, the learning curve. The average complication rate was the total number of complications for the entire case series, and therefore the derivate of the association curve fit to this data set was solved for that average complication rate [13]. Proportion comparisons between groups were performed with Fisher exact test. Statistical significance was taken at 95% confidence interval.

## Results

A total of 132 consecutive MICA procedures have been performed in two and a half years, of this only 100 cases were included in the study while remaining 32 cases were excluded. Of the excluded cases 16 patients had simultaneous second toe correction, 9 cases had other lesser toe corrections and in remaining 7 cases hind foot corrections were also performed. These 100 cases were analysed with a minimum follow-up of 24 months. There were 94 female and 6 male patients with an average age of 50 ± 15 years.

### Operative time

**Fig. 4:**
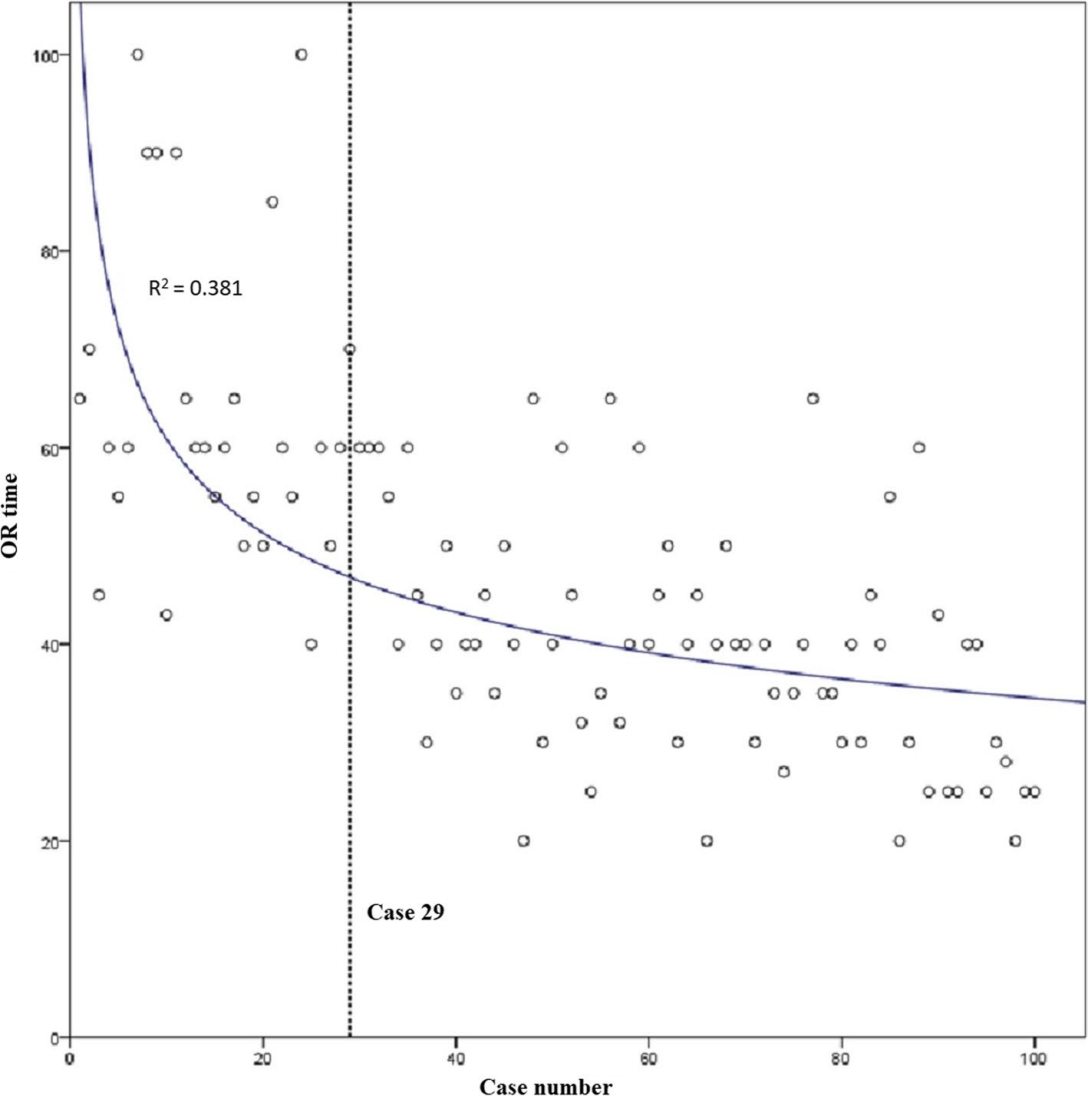
Dissociation curve fit to scatterplot of operative (OR) time. Derivative of the equation solved for overall slope of the data set identifies the learning curve at case 29

The average operative time for all 100 cases was 47 (interquartile range, 35–60) minutes. There was a statistically significant (*p* < 0.001, *r* = -0.669) negative correlation between case number and operative time when examining all cases. A linear slope fit to the overall data set was -0.404 (R^2^ = 0.448). A dissociation curve was fit to the data set (R^2^ = 0.381; Fig. 4), and when solving the derivate of this equation for the case at which the slope of the curve equals -0,404, the result was 29. When cases 1 to 29 and 30 to 100 were compared, the first cohort of cases had a median time of 60 min, and the second cohort of cases had a median time of 40 min, demonstrating a 33% decrease in median time between the first 29 and all subsequent cases.

### Fluoroscopy time, number of scans and exposure

**Fig. 5:**
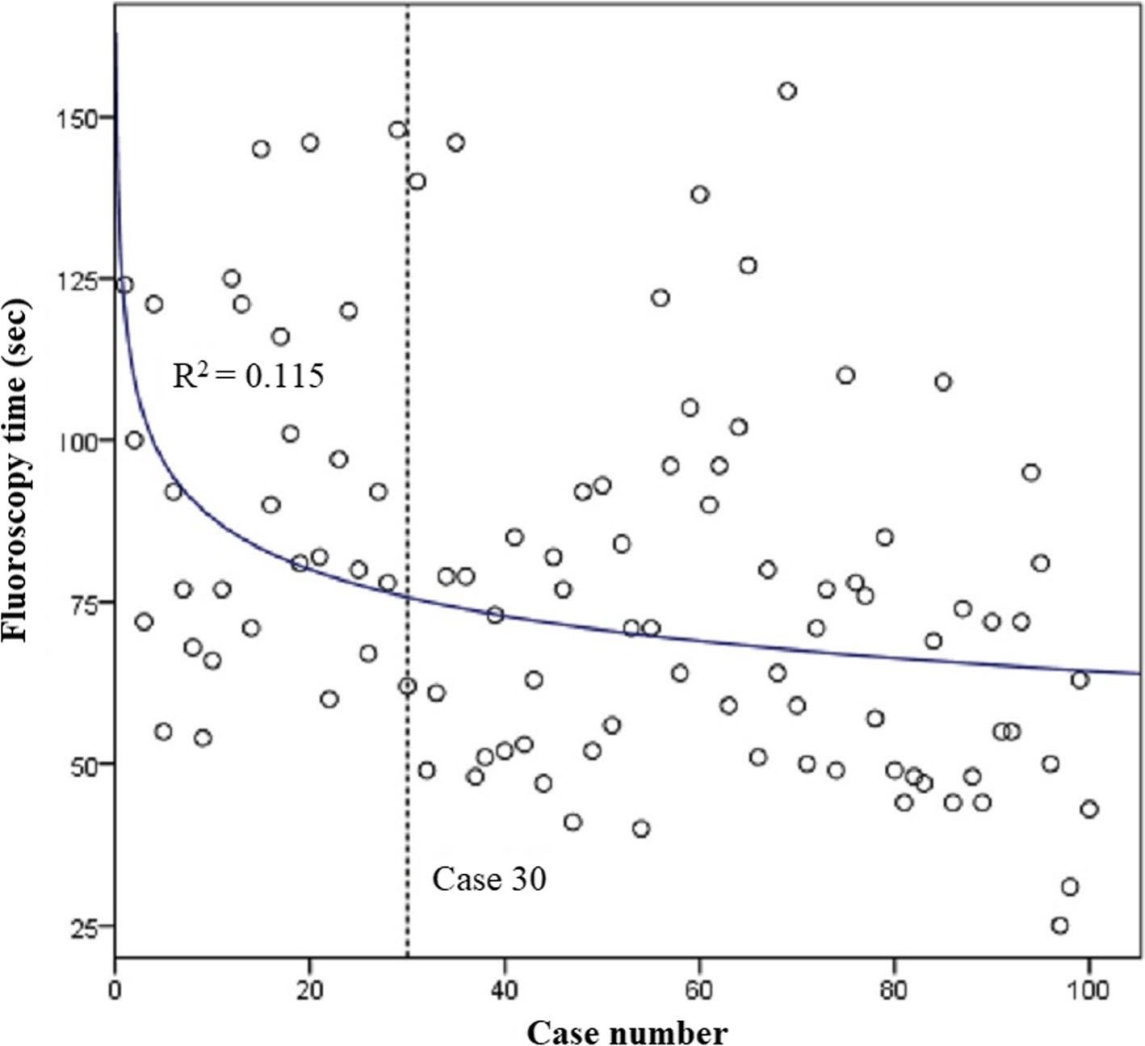
Dissociation curve fit to scatterplot of fluoroscopy time. Derivative of the equation solved for overall slope of the data set identifies the learning curve at case 30

The median fluoroscopy time for all cases was 74 (interquartile range, 55–94) seconds. There was a statistically significant (*p* < 0.001, *r* = -0.363) negative correlation between fluoroscopy time and chronological case number. A slope fit to the overall data set was -0.367 (R^2^ = 0.132). A dissociation curve was fit to the data set (R^2^ = 0.115; Fig. 5), and solving the derivate of this equation for the average rate of change of the model, -0.367, resulted in case 30. Cases 1 to 30 had a median fluoroscopy time of 86 (interquartile range, 71–119) seconds, while cases 31 to 100 had a median fluoroscopy time of 70 (interquartile range 50–85) seconds, a 19% reduction.

**Fig. 6:**
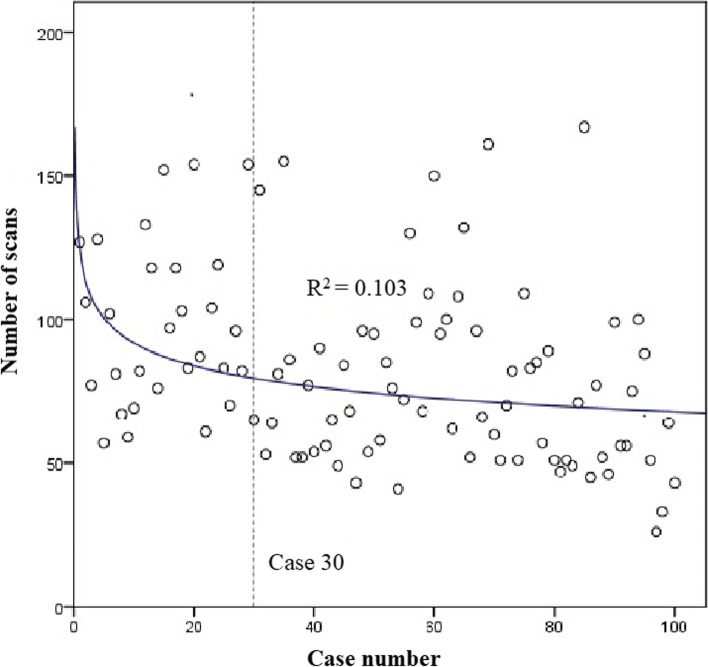
Dissociation curve fit to scatterplot of number of scans taken during the surgery. Derivative of the equation solved for overall slope of the data set identifies the learning curve at case 30

The average number of fluoroscopic scans for all cases was 83 (interquartile range 57–99) scans. There was a statistically significant (*p* < 0.001, *r* = -0.320) negative correlation between case number and operative time when examining all cases. A linear slope fit to the overall data set was -0.351 (R^2^ = 0.102). A dissociation curve was fit to the data set (R^2^ = 0.103; Fig. 6), and when solving the derivate of this equation for the case at which the slope of the curve equals -0.351, the result was 30. When cases 1 to 30 and 31 to 100 were compared, the first cohort of cases had a median of 92 scans, and the second cohort of cases had a median of 69 scans, demonstrating a 25% decrease in median time between the first 30 and all subsequent cases.

The median fluoroscopy total air kerma (TAK) for all 100 cases was 0.60 mGy. There was a difference between the median mGy TAK for cases 1 to 30 (0.67) and cases 30 to 100 (0.57) that was statistically significant (*p* = 0.012).

### Complication rates

**Fig. 7:**
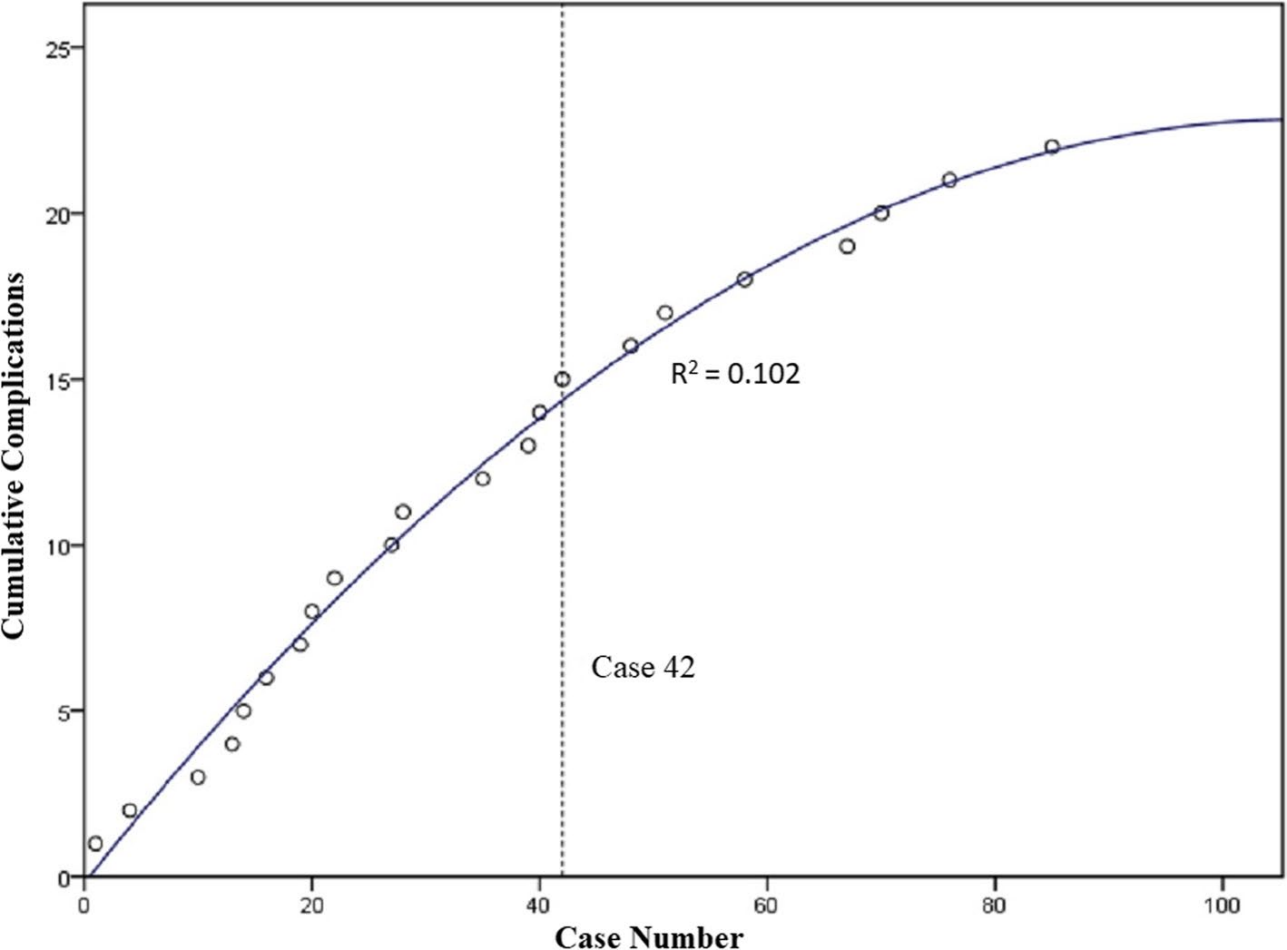
Cumulative number of complications plotted against chronologic case number with association curve fit. Derivate of equation solved for overall complication rate identifies the learning plateau at case 42

A total of 22 complications occurred from 100 cases. A graph of the cumulative number of complications over time displays a plateau starting at case 42, which is where the derivate of the association curve equation equals the overall complication rate of 22% (Fig. 7). Fifteen complications occurred from cases 1 to 42 (36% complication rate) compared to only 7 from cases 43 to 100 (12% complication rate). Therefore, 68% of total complications occurred in the first 42 cases, and this proportion was statistically significant (*p* = 0.007). The 22 complications included 17 hardware removals due to local soft tissue irritation after chevron osteotomy fixation, 2 revision operations due to malposition of the first metatarsal head, 1 stiff first MTP joint, 1 infection and in 1 loss of fixation for chevron osteotomy (Table 1).

**Table 1:** Incidence of complications encountered in the study

Complications	Number
Implant Related	17
Head Malposition	2
MTP Stiffness	1
Infection	1
Loss of Fixation	1

## Discussion

Multiple minimally invasive techniques for hallux valgus correction have been described over the past couple of decades. Bosch et al. in the year 2000 reported good results with subcapital osteotomy technique which was later modified by Gianni et al. where single Kirschner wire stabilization is advocated, but few studies in the literature reported that the technique even though rapid and inexpensive is associated with high complication rate with up to 38% developing hallux valgus recurrence especially because of the lack of stability [14–16]. Later the Reverdin-Isham osteotomy which involved intra-capsular osteotomy, without internal fixation was reported with good results in the early studies [17, 18]. However, the technique was linked to various complications, especially shortening and noncongruence, and hence largely been aborted [6]. Subsequently, Vernois and Redfern described the third generation MICA technique, in which they originally described combination of K-wire and single screw fixation (unicortical) of osteotomy [19]. This was later modified following the reports about the loss of fixation, to the current form of MICA technique where stabilization of the chevron osteotomy is done with 2 screws and bicortical fixation [6, 11, 20–22].

Over last few years, MICA procedure is getting increasingly common because of its added advantages like shorter operation time, smaller scar, and shorter recovery time with outcomes comparable with open surgeries [1, 6, 10, 21]. Many articles have previously stressed out that MICA should not be considered as beginners procedure and preferably needs saw bone and cadaveric specimen training under the guidance of experienced surgeons [21, 23–25]. The learning curve for MICA is significant, and has been studied previously in the literature, however focus was pointed towards deformity correction analysis, postoperative results and complication rates [4, 9, 12]. In this study, we evaluate the learning curve with regard to the duration of radiation exposure in comparison to complications rates, using a reproducible standardized mathematical model.

On analysing the experience of a single surgeon who started performing MICA procedure after completion of a 3-day cadaver lab training, we found that improvement in operative time plateaus after completing 29 cases. This also coincides with the fluoroscopy time as well as the number of exposures taken, that plateaued after 30 cases. In the study by Palmanovich et al. analysing MICA learning curve, the learning curve in regard to number of intraoperative fluoroscopy studies plateaued after the first 27 of 50 cases similar to the present study [9]. In the present study, in terms of complication rates, a much larger learning curve was appreciated, with improvement continuing up until case 42. The complication rate in our study amounted to 22% which is slightly higher than the previous studies it should be noted that 65% of these occurred during the first 42 cases and once the learning curve plateaued the total complication rate was reduced to 12%. It should also be noted that 77% of these complications were hardware related and originated from suboptimal screw position because screw was not screwed deep enough. Therefore, screw head has not been completely submerged under cortex and has irritated soft tissue. Reduction in complication rate was achieved through refinement of technique, greater experience with screw placement, and first metatarsal head positioning after lateral translation, especially in the sagittal plane. Furthermore, complication incidence displays the evolution of the surgeon’s understanding of the procedure in terms of his or her ability to identify and avoid intraoperative risks and optimize each step of the operation to improve outcomes.

In a recent study by Jowett and Bedi, they compared the outcomes of two equal groups, one being the first group of patients (A) and second being the next group of patients (B) to evaluate the learning curve and noticed that the results were better in second group in regard to patient satisfaction, correction and inter-metatarsal angle [4]. Previous studies have also documented that an audit of patient satisfaction and complication rates with self-appraisal will not only improve shortening the learning curve, but also reduces the occurrence of complications [10]. On the other hand, quick achievement of learning plateau in operation time and number of scans taken can give the surgeon a false impression of already being proficient in operation technique. With respect to the findings in our study, understanding of the learning curve may guide the surgeon during the learning process and impose that there is a lag between surgeon feeling comfortable with MICA procedure and an actual decrease in complications rate.

The idea of the learning curve is important for surgeons starting with a new technique, and data collected during the study can serve for representing information to patients about approximate radiation exposure, duration of surgery and information about most frequent complications such as potential necessity to remove screws after procedure. This mathematical and systemic method for defining the learning curve, based on the assumption that proficiency is achieved at the case number where the rate of improvement is lower than the rate of improvement for the entire data, identifies the point at which the surgeon has achieved the majority of improvement to be expected and therefore has become proficient in that particular procedure. Performed study alone does not provide sufficient evidence to precisely define where learning has occurred concerning our chosen variables. However, it is a useful tool to determine where the critical mass for a surgeon to get comfortable with MICA procedure and where his or her skills reach the plateau that provides the lowest risk for complications could be.

This study has several limitations, largely steaming from its retrospective nature, which limited our ability to control sources of bias. The single surgeon nature of the study even though allowed accurate mapping of the learning curve, it also makes generalization of the results more difficult as progression along the curve would vary with the frequency and difficulty of the case load and learning habits of each surgeon. Another drawback of presented learning curve is that it is not applicable as an established norm because it was generated on single surgeons’ limitations. According to authors knowledge such universal learning curve especially tested on larger group of surgeons has not been defined yet. Therefore, presented initiative mathematically supported learning curve could serve as a base for further potential multicentric study performed on several surgeons to generate learning curve that can be presented as a normative.

Mathematical model for curve generation relies on the assumption of fitting a linear slope to a data set and subsequently fitting a nonlinear curve to the data set. If applied to an extensive data set, the linear slope would become very shallow, meaning that the average rate of improvement would decline, thereby skewing the point where proficiency is achieved. Therefore, this model would not be ideal for large data sets. Consequently, it is reasonable to limit or standardize analysed series so it remains focused on that part of the learning curve before the plateau prevails. As a result, the shape of the learning curve reasonably imitates natural logarithmic curve and plateau does not supress beginning of the learning process where radiation exposure is higher and complication rate is more frequent.

Some articles similar to topic of our research have already been published. However, the learning curve for MICA has only once been analysed and represented for operation time and number of snapshots taken. It was performed on half smaller series (50 patients) and has not analysed fluoroscopy time. Because radiation exposure is one of major drawback of MICA, we find it important to analyse it in correlation with learning process. Acquired results can be useful for a surgeon that starts with MICA, because he knows what exposures to expect and how they will drop in the learning process. Nevertheless, learning curve focused on complication rate has already been published and therefore presents minor academic significance but in spite indicates that our data is comparable with other studies and also reproducible especially if experienced foot surgeon familiar with non-minimally invasive hallux correction starts with MICA.

## Conclusions

In our retrospective review of a single surgeon’s learning experience with MICA, we identified that a surgeon might achieve reasonable comfort with the procedure in order to minimize operative time and radiation exposure after approximately 30 cases. However, the plateau is achieved much later for complication rates. These findings represent the highlight of present study and may guide the surgeon during the learning process and impose that there is a lag between the surgeon feeling comfortable with MICA procedure and an actual decrease in complications. Results can be used as supporting information in the educational process for a surgeon choosing to learn MICA and can be valuable when supplying patients with data about the planned procedure. The major drawback of presented mathematical learning curve analysis model is that it can be performed only on limited case series to stay focused on learning process and not on reached plateau. Further potentially multicentric research is reasonable in desire to analyse several surgeons learning curve and to generate a reference learning curve that could serve as a normative.

## Data Availability

The datasets used and/or analysed during the current study are available from the corresponding author on reasonable request.
